# *In-vitro *Effect of Imipenem, Fosfomycin, Colistin, and Gentamicin Combination against Carbapenem-resistant and Biofilm-forming *Pseudomonas aeruginosa *Isolated from Burn Patients

**DOI:** 10.22037/ijpr.2020.111824.13380

**Published:** 2021

**Authors:** Mohammad Yousef Memar, Khosro Adibkia, Safar Farajnia, Hossein Samadi Kafil, Younes Khalili, Robab Azargun, Reza Ghotaslou

**Affiliations:** a *Infectious and Tropical Diseases Research Center, Tabriz University of Medical Sciences, Tabriz, Iran. *; b *Department of Bacteriology and Virology, School of Medicine, Tabriz University of Medical Sciences, Iran. *; c *Research Center for Pharmaceutical Nanotechnology, Tabriz University of Medical Sciences, Tabriz, Iran. *; d *Drug Applied Research Center, Tabriz University of Medical Sciences, Tabriz, Iran. *; e *Immunology Research Center, Tabriz University of Medical Sciences, Tabriz, Iran.*

**Keywords:** Biofilm, Combination therapy, Carbapenem-resistant, Pseudomonas aeruginosa, synergic

## Abstract

The aim of this study was to investigate *in-vitro* antibacterial and antibiofilm effect of colistin, imipenem, gentamicin, and fosfomycin alone and the various combinations against carbapenem-resistant *Pseudomonas aeruginosa* (*P. aeruginosa)*. Eight carbapenem-resistant and biofilm-forming *P. aeruginosa* isolates from burn patients were collected. The mechanisms of resistance to carbapenem were determined by the phenotypic, PCR, and Real-Time PCR assays. The minimum inhibitory concentration (MIC) of antimicrobial agents was determined by the broth micro dilution. To detect any inhibitory effect of antibiotics against the biofilm, the biofilm inhibitory concentration was determined. To detect synergetic effects of the combinations of antibiotics, the checkerboard assay and the fractional inhibitory concentration (FIC) were used. The highest synergic effect was observed in colistin/fosfomycin and gentamicin/fosfomycin (5 of 8 isolates), and the lowest synergic effect was found in gentamicin/imipenem and colistin/gentamicin (1 of 8 isolates). Colistin/fosfomycin, imipenem/fosfomycin, colistin/imipenem, gentamicin/fosfomycin, and gentamicin/imipenem were shown synergic effect for 3, 2, 2, 2 and 1 isolates, respectively. The combination of antibiotics had different effects on biofilm and planktonic forms of *P. aeruginosa*. Therefore, a separate determination of inhibitory effects of the antibiotic in the combination is necessary. Fosfomycin/colistin and fosfomycin/gentamicin were more effective against planktonic form and fosfomycin/colistin against biofilm forms.

## Introduction

*Pseudomonas aeruginosa* is the most frequent cause of healthcare-associated infections in vulnerable patients, particularly in burns cases. *P. aeruginosa *is the most common bacteria isolated from burn infection (1, 2). The frequency of mortality among infected burn patients is three times higher than non-infected burn patients (3). Control of *P. aeruginosa* infections is challenging due to inherently or acquired resistance to different antibiotics and virulence features such as biofilm formation (4, 5). Carbapenems are the most important therapeutic option in the treatment of severe Gram-negative infections. However, the increasing resistance among clinical *P. aeruginosa* isolates has become a major limitation for carbapenem usage (6). The mortality rate of carbapenem-resistant *P. aeruginosa* infection is high, and these isolates are more likely to have cross-resistance to other common antimicrobial agents. *P. aeruginosa* has multifactorial mechanisms of resistance to carbapenem including the production of carbapenemase, reduced or mutation of the outer membrane porin (OprD), decreased levels of the drug accumulation due to the overexpression of efflux-pumps over and the overexpression of AmpC beta-lactamases (7). Biofilms are surface-attached layers of bacteria in an extracellular polymeric compound that may form on human tissues as well as on a diversity of surfaces, such as prosthetic devices, venous catheters, and cardiac pacemakers. Planktonic forms of bacteria are free-living bacteria characterized by un-adhered to surfaces and to each other. Bacteria in a biofilm are much more resistant to antibiotics than to planktonic status. Biofilm formation is one of the most important virulence factors in *P*. *aeruginosa* (8). Biofilm elimination usually needs higher doses and continued drug therapy. However, this often does not successfully eliminate biofilm-caused infections (9). Until now, a new drug has not been introduced to control antibiotic-resistant and biofilm-forming *P. aeruginosa* isolates. It seems unlikely that any new option will be established soon, clinicians may become obliged to administrate old drugs, such as colistin, regardless of their side effects. Therefore, there is a reappearance of attention in the use of combination antimicrobial therapy (10). Combination therapy is one of the most effective strategies for managing the biofilm-forming and antibiotic-resistant* P. aeruginosa *isolates (11, 12). The high frequency of biofilm-forming and resistance of *P. aeruginosa* isolates to wide spectrum antimicrobial agents in burn patients demonstrate that written control programs require treating infection in patients (2, 8 and 10). Combination therapy usually is administrated due to synergic effects to improve the clinical outcome of infections caused by strains that are commonly susceptible to one of the individual antibiotics. Combination therapy also decreases toxicity and might prevent the emergence of antibiotics resistant isolates. The resistance to antibiotics influences the selection of such drugs and their potential for reaching to synergistic effects in combination (8). The combination therapy for *P. aeruginosa *usually includes a β-lactam plus other class of antibiotics. The increasing frequency of carbapenem-resistant may decrease the efficiency of carbapenem in monotherapy and combination therapy against *P. aeruginosa.* Synergy testing provides the evidence of the interaction of two or more antimicrobial agents in combination against bacterial isolates (9, 11). For example, although the fosfomycin is not recommended for the susceptibility testing of *P. aeruginosa* by the Clinical and Laboratory Standards Institute (CLSI), the results of several studies demonstrated that fosfomycin can increase the antimicrobial effects of the antimicrobial agents (13). Because of the impact geographical characteristics on the susceptibility pattern of carbapenem-resistant and biofilm-producing *P. aeruginosa*, the results of such study can be applied in the treatment of *P. aeruginosa* isolates from Tabriz, Iran. The aim of the present study was to evaluate *in-vitro* synergy between some combinations of antibiotics, including imipenem-based combination and others (fosfomycin, colistin, and gentamicin) combinations against planktonic as well as biofilm forms of carbapenem-resistant* P. aeruginosa *isolates (with different mechanisms of carbapenem-resistance) from burn patients.

## Experimental

*Bacterial isolates*

The present study was performed on the carbapenem-resistant and biofilm-forming isolates of *P. aeruginosa*. In this study, forty non-duplicated *P. aeruginosa* isolates were collected from the burn patients and identified by the standard microbiological tests at the Microbiology Department of Tabriz University of Medical Sciences during 2017-2018. The inclusion criteria were carbapenem-resistant and biofilm-forming *P. aeruginosa*. Of 40 isolates, eight carbapenem-resistant, as well as biofilm-forming *P. aeruginosa *was selected. The sample size was determined based on an expected frequency of resistance of 7% (a priori estimate of frequency according to a pilot study result), an accepted error of 4% (required precision of the estimate), and 95% level of confidence, giving a required sample size of 40 samples.

*Mechanisms of resistance to carbapenem*

To screen carbapenem-resistant *P. aeruginosa,* the disk diffusion method was used. The AmpC mediated resistance was detected by the agar plate supplemented with cloxacillin (250µg/mL AmpC β-lactamase inhibitor). At least a twofold decrease in ceftazidime Minimum Inhibitory Concentration (MIC) in the presence of cloxacillin was considered as the AmpC mediated resistance (7). The Phenylalanine-Arginine Beta-Naphthylamide (PaβN) was used as an efflux pump inhibitor at a concentration of 40 μg/mL. The MICs of imipenem and meropenem were determined in the presence of PaβN. At least two-fold reduced MIC in the presence of PAβN considered as efflux pumps mediated resistance (14). Multiplex PCR was performed for the detection of carbapenemase encoding genes. The presence of *bla*_IMP_, *bla*_VIM_, *bla*_SPM_, *bla*_NDM_, *bla*_KPC_ *bla*_OXA-48_, *bla*_BIC_, *bla*_AIM_, *bla*_GIM_, *bla*_SIM_, and *bla*_DIM _was evaluated by the PCR according to a previous study (15). The expression of *mexB* and *oprD* was detected by the real-time quantitative reverse transcription PCR (RT-PCR) and specific primers for *rpsL*, *oprD,* and *mexB* genes as recommended previously (7). The transcription level of r*psL* housekeeping gene was used as standardized expression levels. RNA extraction from bacterial isolates was performed by an RNA extraction kit (SinaClon Co., Tehran, Iran) according to the manufacture guideline. The reactions were carried out using the Rotor-Gene Real-time PCR device (Corbett Research, Sydney, Australia; Model RG 3000) in duplicate runs by the SYBR premix EX TaqII, Tli RNaseH plus (Takara Bio Inc.). Gene’s expression was considered as ratios of the target gene and the housekeeping gene (*rpsL*) according to a relative quantification assay as described previously (7, 14).

*Quantitative detection of biofilm*

The microtiter plate test (MPT) was performed for the quantitative assessment of biofilm formation. Three colonies of the tested organism were suspended in 5 mL of Tryptic Soy Broth (TSB) and incubated for 20 h at 37 °C. After incubation, the culture was vortexed and then diluted 1:100 in TSB supplemented by 0.25% glucose. A 200 µL of this bacterial solution was inoculated in 96 well microplates and incubated for 20 h at 37 °C. The content of the wells was then removed. The wells were carefully washed three times with distilled water and air-dried. The staining was carried by 200 µL of 0.9% crystal violet solution for 15 min. Thereafter, crystal violet was removed, and wells washed with water. The attached crystal violet was solubilized by 95% ethanol and the optical density (OD) of the adherent biofilm was determined twice by the microtiter plate reader at wavelength 450-630 nm. TSB with 0.25% glucose was considered as a negative control (nc). All tested isolates according to a degree of biofilm formation were classified into three groups based on OD value: OD ≤ OD (nc) = non-biofilm producer (-), ODc ≤ 2OD (nc) = weak biofilm producer, 2OD (nc) < OD ≤ 4OD (nc) = moderate biofilm producer, 4OD (nc) < OD = strong biofilm producer (15).

*Antibiotic susceptibility testing*

The antibiotic susceptibility patterns were determined for eight screened carbapenem-resistant and biofilm-forming *P. aeruginosa.* The disk diffusion method was performed according to the CLSI guidelines (17). Antibiotic disks used in this study included ceftazidime, colistin, levofloxacin, cefepime, piperacilin/ tazobactam, aztreonam, ciprofloxacin, and gentamicin. MDR was considered as acquired non-susceptibility to at least one agent in three or more antimicrobial classes (18). The MICs of colistin, imipenem, gentamicin, and fosfomycin were determined by the broth microdilution according to the CLSI guidelines. *Pseudomonas aeruginosa* ATCC 27853 was used as the positive control of antimicrobial susceptibility testing (19).

*BIC determination*

The biofilm inhibitory concentration (BIC) was determined to study the antibiofilm effect of tested antibiotics alone and in the combination. About 100 µg of bacterial suspension equal to 0.5 McFarland in the nutrient broth was transferred to each well of a flat-bottomed 96-well microtiter plate. Biofilm formation was prompted by dipping the pegs of a modified polystyrene microtiter lid into this biofilm growth plate and incubating at 37 °C for 20 h. Peg lids were washed three times in sterile water, placed onto flat-bottomed microtiter plates containing serial concentrations of imipenem, colistin, gentamicin, and fosfomycin only and also in combination with each other in CAMHB per well, and incubated for 20 h at 37 °C. After the incubation time, the peg lid was washed in sterile water and placed into antibiotic-free CAMHB in a flat-bottomed microtiter plate. To transfer biofilms from the pegs to wells, each plate was centrifuged at 805 ×g for 20 min. The peg lid was changed by a usual cover. The OD at 650 nm was determined on a microtiter plate before and after incubation at 37 °C for 6 h. The BIC was defined as the lowest concentration of an antimicrobial agent that lid in an OD 650 variation at or below 10% of the mean of two positive control well readings (21).

*FIC determination*

The antibacterial effects of antimicrobial agent combinations were detected using the checkerboard assay and determination of FICI (Fractional Inhibitory Concentration Index). For the checkerboard assay, each drug’s MIC was determined alone and in combinations against each isolate in one 96-well microplate. A well without antimicrobial agent was considered as control of bacterial growth. The concentration ranges of each antibiotic in combination ranged from 1 to 32 times the MIC. Dilutions of drugs A and B were prepared with a twofold dilution. The FICI was determined as follows:

FICI = MIC of drug A in combination MIC of drug A alone + MIC of drug B in combination MIC of drug B

The FICIs were interpreted as follows: synergy, FICI of ≤0.5, additively FICI of > 0.5 to ≥ 1; no interaction (indifference), FICI of >1 to ≤ 4; antagonism, FICI of >4 (22).

*Statistical analysis*

The data were analyzed using the Statistical Package for the Social Sciences (SPSS) software version 20. A comparison of the data among various groups was performed by Non-parametric tests. *P*-values ≤ 0 .05 were considered as a statistical significance.

## Results

**Figure 1:**
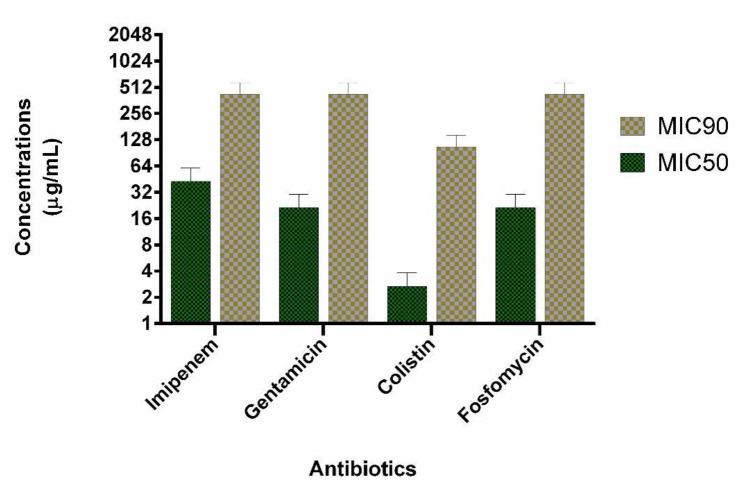
The MIC_50_ and BIC_50 _of tested antibiotics

We evaluated 40 clinical isolates of *P. aeruginosa* collected from burn patients. Among 40 *P. aeruginosa* isolates, 21 (53%) and 25 (63%) were identified as carbapenem-resistant and biofilm-forming isolates, respectively. Among carbapenem-resistant isolates, 13 (62%) were biofilm-forming bacteria. MIC_50_ and MIC _90_ of imipenem were 32 and 32 µg/mL, respectively. Among the biofilm-forming isolates, a strong, moderate, and weak degree of biofilm-forming ability were observed in 15, 6, 4 isolates, respectively. Multifactorial resistance mechanisms were observed in a higher frequency than carbapenem-resistant caused by one resistance mechanism (61.9% *versus* 38.1%). The co-presence of *OprD *decreased expression and *mexB* overexpression were observed as the most common (28.57%) mechanism of carbapenem resistance due to a multifactorial resistance mechanism. AmpC overproduction was detected in 7 (33.3%) isolates. In all AmpC overproduction isolates, they had the other resistance mechanisms such as *OprD* mediated resistance (1 isolate, 4.7%), *mexB* overexpression (4 isolates, 19.1%) and, both *mexB* and OprD mediated resistance (2 isolates, 9.5%). Multifactorial resistance mechanisms were included *OprD* decreased expression (14.28%), the efflux pump overexpression (14.28%) and carbapenemase (9.5%). Table 1 shows the various mechanisms of carbapenem resistance among our isolates. Eight carbapenem-resistant *P. aeruginosa* with different resistance mechanisms and biofilm-forming isolates were selected for the study. Six isolates have a mono-factorial mechanism of resistance to carbapenem and two isolates were resistant due to a multifactorial mechanism. Interestingly, all eight carbapenem-resistant *P. aeruginosa* were MDR. The characteristics and antibiotics resistance profile of the selected isolates are shown in Table 2. The MICs of colistin, imipenem, gentamicin, and fosfomycin are shown in Figure 1. The highest antimicrobial synergic effects (FICI ≥ 0.5) have been detected for the combination of colistin/fosfomycin and gentamicin/fosfomycin (5 of 8 isolates). Whiles lowest synergic effect against the planktonic form of bacteria has been observed in the case of gentamicin/imipenem and colistin/gentamicin (1 of 8 isolates). The antagonism effect was not observed in the present study. There was no significant statistical association between the synergistic effects of drug combinations against the planktonic form with the type of resistance mechanisms to carbapenems. Table 3 shows the antimicrobial effect of different antibiotics alone and in the combination against the planktonic form of bacteria. The antibiofilm effect of antibiotics was tested alone or in combination against biofilm-producing isolates. None of the antibiotics alone eradicate biofilm form at MIC or sub-MIC of each antibiotic agent. Figure 1 presents the MIC_50_ and BIC_50_ of antibiotics. In comparison, colistin/fosfomycin, imipenem/fosfomycin, colistin/imipenem, gentamicin/fosfomycin, and gentamicin/imipenem showed a synergic effect for 3, 2, 2, 2, and 1 isolates, respectively. There was no significant statistical association between the synergistic effect of antibiotics combination against bacterial biofilm and the ability of biofilm formation (strong, moderate, and weak types). The combination of gentamicin/colistin did not show any synergistic effect. Table 3 shows the BIC of antibiotics against the biofilm form of bacteria.

## Discussion

The emergence and distribution of carbapenem-resistant strains may considerably compromise their usefulness (10). The results of our study showed a high prevalence of carbapenem-resistant (52.5%) *P. aeruginosa* isolates among burn patients. Another study from Iran also reported a high prevalence (94.7%) of carbapenem-resistant *P. aeruginosa* isolates from burn patients (23). The frequency of carbapenem-resistant *P. aeruginosa* is significantly different among various settings due to differences in infection control, antibiotic use, geographic area, and the various treatment procedures (16). Multifactorial carbapenem resistance mechanisms were detected in the higher frequency than carbapenem resistance caused by one mechanism. In the present study, the most frequent mechanism of resistance to carbapenems was the overexpression of efflux pumps detected phenotypically or genetically among 71.4% of isolates. The overexpression of efflux pumps has been reported as the most common mechanism of resistance to carbapenems among *P. aeruginosa* (7, 24).

Antimicrobial combination therapy is one of the most effective options for the control of resistance to antibiotic isolates. Synergy assessment has clarified the interaction of two drugs in combination against bacterial isolates. In the present study, the interactions of imipenem, colistin, fosfomycin, and gentamicin have been evaluated for the eradication of planktonic and biofilm forms of carbapenem-resistant *P. aeruginosa* isolates *in-vitro* condition. According to the microbroth dilution, 4 of 8 isolates were colistin susceptible (MIC ≤ 2 µg/mL) and 4 of 8 isolates were colistin intermediate (2 µg/mL < MIC < 8 µg/mL). The emerging of colistin-resistant *P. aeruginosa* isolates has been reported in other studies (8, 25). Colistin is commonly considered as the last resort for infections caused by carbapenem-resistant *P. aeruginosa* isolates. In clinical practice, the combination therapy is commonly used to increase its antibacterial effect, despite the consequent increase in toxicity (26). In the present study, the combination of colistin and fosfomycin showed the synergistic and additive effect against 5/8 and 2/8 isolates, respectively. The synergic effect of colistin and fosfomycin was observed among 3 colistin intermediate isolates. *In-vitro* activity of fosfomycin in combination with colistin against carbapenem-resistant Gram-negative bacteria has been evaluated by other studies. Similar to our study, Di *et al.* reported the combination of colistin with fosfomycin had a synergistic and partial synergistic effect in 49.43% of the isolates, and no antagonism was observed (27). Some studies have also reported that fosfomycin improves the treatment outcomes, prevents antimicrobial resistance, and decreases the toxicity induced by the different antibiotics (28). Souli *et al.* found 5 mg/L colistin combined with 100 mg/L fosfomycin resulted in a bactericidal effect against 65% of carbapenem-resistant *Klebsiella* *pneumonia* isolates (29). In combination, the fosfomycin and colistin MIC for most of the isolates were significantly lower than the plasma concentrations that can be achieved for both agents (30). The combination of fosfomycin with colistin has been studied in infections due to the foreign-body model and is suggested as a promising treatment option for implant-associated infections by Gram-negative bacilli (31). According to the results of the checkerboard method, fosfomycin plus gentamicin has a synergistic effect against 5 of 8 isolates. Okazaki *et al.* reported that fosfomycin in combination with gentamicin had an efficacy rate of 70% against MDR *P. aeruginosa* isolates (32). Fosfomycin may increase the cellular uptake of aminoglycosides, resulting in increased inhibition of protein synthesis and ultimately bacterial killing (13). The synergy effect of aminoglycosides plus fosfomycin not only showed *in-vitro* but also increased the therapeutic effect in a rat model. This combination offers an effective treatment strategy against some drug-resistant bacteria (21). In the present study, the combination of fosfomycin and colistin, fosfomycin and imipenem showed a significant synergy (50%) and additive effect (50%) against the planktonic forms of imipenem-resistant *P. aeruginosa*. The *in-vitro* effects of fosfomycin in combination with other antibiotics have been studied against clinical isolates of *P. aeruginosa* with different antibiotic resistance patterns in the different studies. According to the results of Okazaki *et al*., fosfomycin plus carbapenems had an appropriate efficacy rate (76.6% with meropenem and 73.3% with imipenem) against MDR *P. aeruginosa *(32). In contrast, Tessier reported that fosfomycin/imipenem had an additive and indifference effect on 37% and 63% of isolates, respectively (34). Samonis *et al.* reported the synergic effect for the combination of fosfomycin with imipenem and meropenem on 46.7%, 53.3% of isolates, respectively (35). It is speculated that fosfomycin may offer alternative permeability routes for antibiotics into the bacteria by destroying the outer membrane and increases the antimicrobial effects (32,33). Therefore, even if an MDR *P. aeruginosa* isolate is highly resistant to an antibiotic, it may be sensitive to the same antibiotic when administered in combination with fosfomycin. Antibiotic combinations must be carefully considered to minimize the selection of strains with double resistance. It has been shown that the probability emergence of mutants resistant to the combination of fosfomycin with imipenem is significantly high but is not detectible for combinations of fosfomycin with tobramycin, amikacin, meropenem, ciprofloxacin, and colistin (35).

In this study, the imipenem and colistin combination has a synergic or additive effect against 3 and 4 isolates, respectively. The synergistic or additive effect of colistin plus imipenem was previously reported against imipenem-resistant and colistin-resistant subpopulations of *P. aeruginosa *(38). The combinations of gentamicin/colistin and gentamicin/imipenem had synergic for 1/8 of isolates. The synergistic effect of imipenem in the combination with aminoglycosides has been reported for 10% of imipenem-resistant and 8% of MDR *P. aeruginosa *isolates (39, 40). Considering the increased prevalence of multidrug-resistant organisms, synergism testing becomes a potentially powerful tool to help in the selection of appropriate antibiotic therapy (41). *P. aeruginosa*, the form of biofilms, is significantly resistant to eradication by antibiotic therapy. Conventional antibiotic susceptibility testing surveys the efficiency of antibiotics against the planktonic form of organisms under aerobic conditions. Thus, the determination of an antibiotic’s BIC may help treat infections caused by biofilm-producing bacteria (8). None of the imipenem, colistin, gentamicin, and fosfomycin was inhibited biofilm formation alone at MIC or sub MIC of each antibiotic agent in the present study. A synergetic or additive effect was detected between colistin/fosfomycin, imipenem/fosfomycin, gentamicin/fosfomycin, gentamicin/imipenem, and imipenem/colistin. For colistin/gentamicin, only the additive effect was observed in 5 of 8 isolates. Other studies have shown the synergistic effect of the various antibiotics for biofilm eradication. However, due to the high level of different antibiotics BIC, even with the potential of synergistic interactions, the use of these antibiotics may be associated with the toxicity effect in the patients. While synergistic interaction between several combinations was observed in the present study, clinical observation to support these results may be conflicting. In some studies, a significant association between *in-vitro* synergy assessment (by the time-kill or checkerboard assay) and the clinical outcome was not found. Additionally, the results of the checkerboard synergy test may not be correlated with the other method. Therefore, the results of the present study are better to be confirmed by others *in-vitro* synergy testing and clinical studies.

**Table 1:** The various mechanisms of resistance to carbapenem in 21 carbapenem-resistant *P. aeruginosa *isolated from the burn patients according to monofactorial resistance mechanisms or multifactorial resistance mechanisms

**Mechanism of Resistance**		**Number (%)**
Monofactorial	Carbapenemase	2 (9.5%) (one for *bla*_IMP_ and one for *bla*_VIM_)
	OprD	3 (14%)
	Efflux Pump	3 (14%)
Multifactorial	OprD/AmpC	1 (4.7%)
	Efflux Pump/AmpC	4 (19.5%)
	OprD/Efflux Pump	6 (28.5%)
	OprD/Efflux Pump /AmpC	2 (9.5)

**Table 2.** The different resistance mechanisms and antibiotics resistance profile in 8 carbapenem and biofilm-forming isolates

**Isolates**	**Mechanism of carbapenem-resistant**	**Degree of biofilm formation**	**Resistant to antibiotics**
1	Carbapenemase	S	CAZ, FEP, LVX, AZT, CIP, GEN
2	Carbapenemase	S	CAZ, LVX, CIP, GEN
3	Efflux Pump	W	CAZ, FEP, LVX, PTZ, AZM, CIP, GEN
4	Efflux Pump	S	CAZ, FEP, AZM, GEN
5	OprD	W	FEP, LVX, , PTZ, AZM CIP
6	OprD	S	CAZ, FEP, LVX, PTZ, AZM, CIP, GEN
7	OprD/Efflux Pump	M	CAZ, FEP, LVX, PTZ, AZM, CIP
8	Efflux Pump/AmpC	M	FEP, LVX, PTZ, AZM, CIP

S: strong, M: moderate, W: weak, CAZ: ceftazidime, FEP: cefepime, LVX: levofloxacin, PTZ: piperacilin/tazobactam, AZM: aztreonam, CIP: ciprofloxacin, GEN: gentamicin.

**Table 3:** The MICs and BICs of antibiotics alone and the combination against biofilm and planktonic form of tested bacteria

**Isolates,** **n**	**Inhibitory concentration of tested antibiotics**								**The combination effects of tested antibiotics**												
	**FOS** **(µg/mL)**		**IMI** **(µg/mL)**		**COL** **(µg/mL)**		**GEN** **(µg/mL)**		**COL/FOS**		**IMI/FOS**		**GEN/FOS**		**GEN/IMI**		**IMI/COL**		**COL/GEN**		
	**BIC**	**MIC**	**BIC**	**MIC**	**BIC**	**MIC**	**BIC**	**MIC**	**Bio**	**Pln**	**Bio**	**Pln**	**Bio**	**Pln**	**Bio**	**Pln**	**Bio**	**Pln**	**Bio**	**Pln**
1	512	128	512	32	64	2	1024	32	A	S	S	S	NI	S	A	A	NI	A	NE	S
2	512	64	512	64	128	4	512	128	NI	S	A	S	S	S	NI	A	NI	A	A	A
3	1024	32	256	16	128	2	512	256	NI	NE	NI	A	NI	A	NI	A	A	S	NE	A
4	1024	64	256	16	64	1	1024	16	A	A	NI	A	NI	A	NI	S	S	A	A	A
5	1024	256	512	64	128	4	512	4	S	S	S	S	A	S	NI	A	NI	S	A	NI
6	1024	32	1024	128	128	4	512	32	NI	S	NI	S	NI	S	NI	A	NI	NI	NI	A
7	512	16	256	32	128	2	512	4	S	S	NI	A	NI	S	S	A	S	A	A	A
8	512	64	512	32	128	4	512	8	S	A	NI	A	S	NI	NI	A	NI	S	A	A

A: additive; BIC: Biofilm Inhibitory Concentration; Bio: biofilm; COL: colistin; GEN: gentamicin; IMI: imipenem; MIC: Minimum Inhibitory Concentration; N: number; NI: no-interaction; Pln: planktonic; S: synergic; FOS: fosfomycin.

## Conclusion

The results of the present study show that the combination of antimicrobial agents has different effects on biofilm and the planktonic forms. However, a separate determination of inhibitory effects of the antibiotic in the combination against the planktonic and biofilm may be applicable for guiding antibiotic therapy. The combination therapy using fosfomycin and colistin, gentamicin, or imipenem may be helpful in eradicating the planktonic form of carbapenem-resistant *P. aeruginosa*. The high concentrations of colistin/fosfomycin, imipenem/fosfomycin, gentamicin/fosfomycin, gentamicin/imipenem, and imipenem/colistin combinations in biofilm-forming bacteria show the synergistic effect.
